# Effects of Repeated Thermo-Mechanical Processing on the Degradation Behavior of Bottle-Grade PET Under Controlled Conditions

**DOI:** 10.3390/polym18030416

**Published:** 2026-02-05

**Authors:** Mária Straková, Slávka Hlaváčiková, Jozef Feranc, Henrieta Suchánková, Zuzana Kramárová, Michal Ďurfina, Leona Omaníková, Mohammadhassan Rahnama Hezaveh, Katarína Tomanová, Zuzana Vanovčanová, Ján Kruželák, Pavol Alexy, Roderik Plavec

**Affiliations:** Institute of Natural and Synthetic Polymers, Faculty of Chemical and Food Technology, Slovak University of Technology, Radlinského 9, 812 37 Bratislava, Slovakia; maria.strakova@stuba.sk (M.S.); slavka.hlavacikova@stuba.sk (S.H.); jozef.feranc@stuba.sk (J.F.); xsuchankovah@stuba.sk (H.S.); zuzana.kramarova@stuba.sk (Z.K.); michal.durfina@stuba.sk (M.Ď.); leona.omanikova@stuba.sk (L.O.); mohammadhassan.hezaveh@stuba.sk (M.R.H.); katarina_tomanova@stuba.sk (K.T.); zuzana.vanovcanova@stuba.sk (Z.V.); jan.kruzelak@stuba.sk (J.K.);

**Keywords:** material recycling, polyethylene terephthalate, rheology, molecular characteristics, mechanical properties

## Abstract

Mechanical recycling of polyethylene terephthalate (PET) is a key strategy for circular packaging applications; however, repeated thermo-mechanical processing leads to progressive polymer degradation. In this study, the effect of controlled repeated extrusion on the degradation behavior of bottle-grade PET was systematically investigated under laboratory conditions. Mechanical recycling was simulated using a co-rotating twin-screw extruder, where PET was subjected to up to four consecutive processing cycles corresponding to a cumulative residence time of 8 min. Progressive processing resulted in chain scission, reflected by a decrease in intrinsic viscosity from approximately 0.80 to 0.65 dL·g^−1^ and a corresponding reduction in molecular weight. Melt flow rate increased accordingly, indicating a gradual loss of melt strength. Differential scanning calorimetry revealed that the glass transition and melting temperatures remained nearly unchanged, while the degree of crystallinity increased from approximately 23.0% to 29.5%, accompanied by changes in crystallization behavior. These structural changes led to reduced ductility, with elongation at break decreasing from about 84% to 60%. Optical analysis showed systematic material darkening, and a strong linear correlation between lightness (L*) and intrinsic viscosity was observed. By isolating intrinsic thermo-mechanical degradation effects under controlled processing conditions, this study enables a clearer definition of realistic reuse limits for mechanically recycled bottle-grade PET. The results indicate that bottle-grade PET retains properties compatible with demanding applications only after a limited number of thermo-mechanical processing cycles, whereas further processing restricts its usability to less demanding applications such as fibers, films, and non-food packaging.

## 1. Introduction

The continuous increase in plastic waste generation is a critical global challenge for ecology and human health, requiring targeted solutions from society to reduce pervasive plastic pollution [1,2,3,4,5]. Global plastic production amounts to hundreds of millions of tons of new materials each year, influencing both the world economy and the environment [6]. Of the total amount of plastics used, only about 15% is recycled and another 25% is recovered for energy. As a result, around 220 million tons of plastic waste are generated annually on a global scale, with a large portion ending up in landfills or the natural environment [7]. Plastic recycling is essential to prevent the excessive accumulation of polymeric materials in the environment after their service life, thereby reducing pollution [5,8,9,10,11].

In the packaging industry, 33% of the total plastic production volume is used. Polyethylene terephthalate (PET) is one of the most widely used polymers in this sector [12,13,14,15]. It ranks among the most extensively utilized plastic materials, with the annual global production of PET polymer exceeding 80.9 million tons in 2021. It is projected to reach 114.7 million tons by 2028, with an annual growth rate of 5.2% [16,17].

Polyethylene terephthalate (also poly(ethylene benzene-1,4-dicarboxylate)) is a semi-crystalline thermoplastic polymer material widely used in everyday life [18,19]. It belongs to a group of polymeric materials suitable for beverage packaging, especially for soft drinks and mineral waters—mainly because of its low weight, excellent transparency, barrier properties, impact toughness, and strength characteristics [20,21,22,23]. It is also resistant to a wide range of chemicals, including acids, bases, and solvents, which makes it suitable for manufacturing containers and packaging for the pharmaceutical industry [24,25,26].

Currently, approximately 66% of PET material intended for recycling is collected within the European Union. The remaining portion is incinerated or landfilled, resulting in the release of greenhouse gases. Conversely, a higher PET recycling rate would enhance the circular economy and reduce CO_2_ emissions during the production and processing of “virgin” PET polymer material into final products [12,27]. Despite extensive research on PET recycling, the influence of controlled thermo-mechanical processing history on the functional limits of bottle-grade PET remains insufficiently quantified.

Starting from 2025, manufacturers of PET plastic packaging are required to place on the market only PET bottles containing at least 25% recycled material. Another change regarding the minimum recycled plastic content for beverage bottles is expected in 2030, when all plastic beverage bottles will be required to contain at least 30% recycled plastic, calculated as an average for all beverage bottles placed on the market on the territory of the member state [28,29,30]. The European legislation has mandated that all member states achieve a 90% collection rate of PET plastic beverage containers by 2029. The deposit return system for single-use beverage packaging is based on the principle of a circular economy, which enables the collection of larger quantities of higher-quality material for recycling, reuse in the production of new packaging, and the conservation of natural resources. In Slovakia, the Deposit Return System for single-use beverage containers was introduced on 1 January 2022. From its launch until the end of 2024, more than 3.5 billion beverage containers were collected in Slovakia, 57% of which were PET beverage bottles [22,31,32]. The annual deposit return rate in Slovakia is approaching 90%, bringing the country close to the target set by European legislation 2019/904 [28].

Recycling of PET represents one of the most successful and widespread examples of polymer recycling. The main driving force behind the increasing rate of post-consumer PET recycling is its extensive use, particularly in the packaging industry [33], which has made PET the primary target for plastic recycling [34]. Compared to the production of virgin PET, recycling can significantly reduce greenhouse gas emissions—life cycle assessment (LCA) studies have shown that PET recycling can reduce energy consumption by up to 84% and greenhouse gas emissions by 71%, with lower overall energy demand and carbon footprint compared to virgin PET production [24,35].

**Figure 1:**
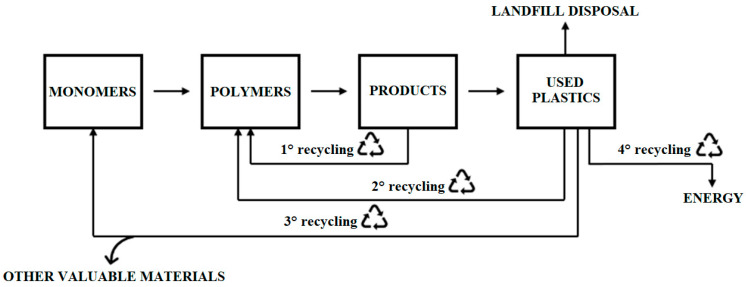
Types of plastic waste recycling [38].

The processing and recycling of polymer waste can be divided into several main categories (Figure 1): primary recycling (re-extrusion), secondary recycling (mechanical recycling), tertiary recycling (chemical recycling), and quaternary recycling (energy recovery) [36,37].

In the mechanical “bottle-to-bottle” recycling process, post-consumer PET bottle waste is processed after being delivered to recycling facilities in the form of compressed PET bottle bales. These bales then undergo a multi-stage recycling process [39]. The multi-stage process includes sorting and separation of waste, grinding and crushing into PET flakes, washing to remove impurities and contaminants, drying, and finally reprocessing the rPET without altering its chemical composition to produce polymer pellets and granules that serve as a substitute for virgin plastics on the market [40,41]. Subsequently, rPET materials are blended with virgin PET, and through injection stretch blow molding technology, preforms are produced, which are then used to manufacture new PET bottles [42].

The main challenges associated with recycled plastics are various contaminants present in plastic waste, reduction of molecular weight during recycling steps, and degradation caused by oxygen, light, temperature, or water throughout their lifespan [43,44,45]. The intrinsic viscosity of PET materials significantly decreases during recycling and processing due to degradation processes (thermal and hydrolytic), which lead to product degradation and a loss of properties [46].

Multiple studies have confirmed that PET recycling, including the use of rPET in beverage bottles, is well established and that repeated recycling loops are technically feasible [42,44,47,48,49]. However, they consistently emphasize that the final quality of rPET strongly depends on proper control of processing parameters and the quality of the collected material. Among the most critical issues are color changes, especially yellowing caused the formation of chromophoric structures during thermal exposure, and shifts in IV values, which are essential for bottle-grade PET [44,47,50,51].

Despite these risks, research also shows that rPET can maintain quality comparable to virgin PET over numerous cycles. In several long-term recycling evaluations, rPET showed no significant loss of mechanical properties or accumulation of monitored substances and was found to maintain quality standards suitable for food-grade bottle production even after eleven cycles, highlighting its potential for ongoing circular use when input material quality and process parameters are properly controlled [44,48].

Other studies, however, point to certain limitations. Repeated processing was found to cause chain scission, reduced molar mass and viscosity, increased brittleness, and processing difficulties. Increased crystallinity and altered crystallization temperature indicate that the thermomechanical history of rPET significantly affects its molecular structure. Further studies indicate that rPET exhibits thermal properties different from virgin PET, which may influence its processability [8,48,51,52,53].

Overall, extensive research confirms that repeated PET recycling is achievable without major loss of mechanical performance, but challenges remain in color stability, processability, and controlling chain degradation [44,47,48].

Despite extensive research on PET recycling, a clear understanding of how cumulative thermo-mechanical stress during repeated processing affects the usability limits of bottle-grade PET remains incomplete. Many studies on recycled PET focus on heterogeneous post-consumer materials, where the combined effects of contamination, variable feedstock quality, and processing conditions complicate the interpretation of degradation mechanisms. In contrast, systematic investigations under controlled laboratory conditions are essential to isolate intrinsic thermo-mechanical degradation and to establish realistic processing-related property limits for bottle-grade PET.

In this work, mechanical recycling of PET was simulated under well-defined laboratory conditions using repeated extrusion with precisely controlled residence time. The study systematically correlates molecular, rheological, thermal, optical, and mechanical changes occurring during successive processing cycles, enabling a comprehensive assessment of degradation mechanisms and their impact on material performance. Particular attention is paid to the relationship between intrinsic viscosity and optical properties as a potential rapid indicator of material quality. The results provide experimentally supported limits for the reuse of mechanically recycled PET in bottle applications and offer guidance for its further utilization in a circular economy context.

## 2. Experimental

### 2.1. Materials

Bottle-grade polyethylene terephthalate (PET) supplied in pellet form by UAB NEO GROUP (Rimkai, Lithuania), marketed under the trade name NEOPET 80 and conforming to standard food-contact PET resin specifications, was used as the virgin material (vPET). The selected grade exhibited an intrinsic viscosity of approximately 0.80 dL·g^−1^, which is typical for PET materials intended for beverage packaging and bottle-to-bottle recycling applications.

The use of virgin PET as the starting material enabled the isolation of intrinsic thermo-mechanical degradation effects, eliminating the influence of contaminants, additives, and heterogeneous feedstock commonly present in post-consumer recycled PET. This approach allowed the systematic evaluation of property changes arising solely from controlled processing history.

Prior to each processing step, the material was dried in a vacuum oven at 160 °C for 5 h under reduced pressure (<0.3 atm) in order to minimize moisture-induced hydrolytic degradation during melt processing. Under these conditions, drying is not expected to cause significant thermal degradation of PET, but rather ensures that the observed changes in molecular, thermal, optical, and mechanical properties can be primarily attributed to thermo-mechanical stress during extrusion.

### 2.2. Processing and Experiments

#### 2.2.1. Multiple Extrusion

In the laboratory, the process of mechanical recycling of PET material was simulated using a laboratory co-rotating twin-screw LTE 16-52, equipped with a screw of 16 mm in diameter and L/D ratio = 52. The repeated extrusion approach was selected to simulate successive mechanical recycling steps under controlled laboratory conditions, where cumulative melt residence time represents a key parameter governing thermo-mechanical degradation of PET. Head of extruder was equipped with the circular cross section die nozzle, diameter 2 mm. The temperature profile of the extruder was 5 × 275–2 × 280–3 × 270–2 × 260–250 °C in the direction from hopper to head and the screw speed was 100 min^−1^. The polymer material was repeatedly subjected to thermo-mechanical stress by passing through the twin-screw extruder. The total number of processing cycles through the extruder was four. The residence time of the polymer material in the processing device during each of the four processing passes was 120 s (2 min). The polymer melt exiting the extruder in the form of a strand was subsequently air-cooled on a conveyor belt and pelletized. From each extrusion cycle, a representative sample was collected for the determination of molecular, rheological, thermal, physico-mechanical, and colorimetric properties. The designation of each studied sample is stated in Table 1. The processing time reported in Table 1 corresponds to the cumulative residence time of the material in the extruder resulting from successive processing passes. By maintaining identical processing conditions for each extrusion pass, the influence of cumulative thermo-mechanical stress could be evaluated independently of variations in processing parameters.

All tested properties except molecular characteristics were performed in multiple replicates, and the reported values represent mean values.

Samples collected after each processing cycle enabled a systematic correlation between cumulative processing history and changes in molecular, thermal, optical, and mechanical properties.

#### 2.2.2. Molecular Characteristics Measurement

Molecular characteristics were investigated in order to directly assess degradation-induced changes in polymer chain length resulting from repeated thermo-mechanical processing. Gel permeation chromatography (GPC) was selected as a suitable technique to monitor variations in molecular weight distribution associated with chain scission during successive extrusion cycles. To determine the molecular weight and distributions of molecular weight of the PET samples, gel permeation chromatography (GPC) was used. The samples were dissolved in a solvent mixture of dichloromethane and 1,1,1,3,3,3-hexafluoro-2-propanol in a ratio of 95/5 vol.% with a flow rate set at 1 mL/min. For the analysis, 10 mg of each PET sample was dissolved in 1 mL of eluent. The separation of the sample components was carried out using a PL gel 5 μm MIXED-B column with dimensions of 300 × 7.5 mm. Detection was performed with a SEDEX LT-ELSD Model LC detector, (SEDERE, Alfortville, France) using nitrogen as the carrier gas at an evaporation temperature of 50 °C. The resulting chromatographic data were evaluated based on calibration curve obtained from PMMA standards with molecular weights ranging from 1900 to 612,000 g/mol.

#### 2.2.3. Intrinsic Viscosity and Melt Flow Rate Index Measurement

Intrinsic viscosity and melt flow rate were evaluated to characterize changes in melt rheological behavior associated with thermo-mechanical degradation of PET. While intrinsic viscosity provides an indirect measure of molecular weight, melt flow rate offers complementary information on processability and flow behavior during repeated melt processing. The determination of intrinsic viscosity (IV) and the melt flow rate index (MFR) was carried out according to standard STN ISO 1133. The intrinsic viscosity values were determined indirectly from melt flow rate measurements using an instrument-specific calibration curve, which is commonly applied for comparative evaluation of PET degradation trends. The measurements were performed using a Dynisco Polymer Test LMI 4001 DE plastometer, (Dynisco, Franklin, MA, USA) under the following test conditions: the test temperature was set to 285 °C, with a preheating time of 300 s, while a load of 2.16 kg was applied. The melt was forced through a capillary die with a diameter of 2 mm and a length of 8 mm. IV values were used for comparative trend analysis and correlated with independently determined molecular weight data.

#### 2.2.4. Thermophysical Properties Measurement

Thermal properties were analyzed to evaluate the influence of repeated thermo-mechanical processing on phase transitions and crystallization behavior of PET. Differential scanning calorimetry (DSC) was employed as a suitable technique to detect changes in thermal transitions and degree of crystallinity associated with degradation-induced modifications of polymer chain structure. The measurement of thermophysical properties was carried out using a differential scanning calorimeter (Mettler-Toledo Inc., Greifensee, Switzerland). The basic thermophysical properties such as glass transition temperature, crystallization temperature and melting temperature of samples were measured after each processing cycle. Thermal properties were evaluated from the second heating and cooling scans to eliminate thermal history effects.

The conditions for DSC measurements are summarized in Table 2. Nitrogen was used as the inert gas, and data were evaluated using the SW STARe, version 17.0 (Mettler Toledo, Greifensee, Zurich, Switzerland).

In addition to thermal property measurements, the degree of crystallinity ($\chi_{c}$) was determined. The degree of crystallinity ($\chi_{c}$) was calculated according to Equation (1). In Equation (1), ΔHm represents the melting enthalpy of the PET sample determined from the second heating scan, while ΔH_0_ corresponds to the melting enthalpy of 100% crystalline PET, taken as 140 J·g^−1^. The degree of crystallinity ($\chi_{c}$) was calculated as the ratio of the measured melting enthalpy to the reference enthalpy of fully crystalline PET.

(1) $$\chi_{c}=\frac{\Delta H_{m}}{\Delta H_{0}}\times 100\%$$

#### 2.2.5. Color Changes Measurement

Color changes were evaluated to assess the influence of repeated thermo-mechanical processing on the optical appearance of PET, which is a critical quality parameter for packaging applications. Colorimetric analysis based on the CIE L*a*b* system was employed as a sensitive, non-destructive method to detect degradation-related changes associated with the formation of chromophoric structures. The color of the multiple-recycled PET samples was measured using a Techkon–SpectroDens spectrophotometer,(TECHKON, Königstein im Taunus, Germany) according to the CIE Lab color scale. Measurements were performed under illuminant D65 with a 10° standard observer angle, without the use of a polarizing filter. A white standard of (100.0; 0) was applied for calibration, and the measurement aperture diameter was set to 3 mm.

From the trichromatic coordinates, the total color difference was calculated according to Equation (2). In Equation (2), L* represents the lightness coordinate, while Δa* and Δb* denote the differences in the red–green and yellow–blue color coordinates, respectively, used to calculate the total color difference (ΔE) of the samples relative to the white standard.

(2) $${\Delta E}_{a,b}^{*}=\sqrt{{\left(\Delta L*\right)}^{2}+{\left(\Delta a*\right)}^{2}+{\left(\Delta b*\right)}^{2}}$$

#### 2.2.6. Mechanical Properties Measurement

Mechanical properties were evaluated to assess the influence of repeated thermo-mechanical processing on the deformation behavior and ductility of PET. Tensile testing was selected as a suitable method to capture changes in mechanical response associated with molecular degradation and increased crystallinity. Tensile tests were performed using Zwick/Roell testing machine (ZwickRoell, Ulm, Germany) in accordance with STN ISO 527, at cross-head speed 1 mm/min in the deformation range of 0–3%. When deformation reaches value 3% of elongation, the cross-head speed increased up to 50 mm/min. The samples were in form of strands from each extrusion cycle with a circular cross-section with a diameter of 2.0 mm. Tensile strength at break (σb) and the elongation at break (εb) were determined. All tensile tests were performed on five specimens per material, and the reported values represent mean values.

## 3. Results and Discussion

This section presents a comprehensive evaluation of the effects of repeated thermo-mechanical processing on bottle-grade PET under controlled laboratory conditions. Mechanical recycling was simulated through successive extrusion passes in a co-rotating twin-screw extruder, allowing the polymer material to experience a systematically increasing cumulative residence time in the melt.

This experimental approach enables the isolation of intrinsic degradation phenomena associated with thermo-mechanical stress, while minimizing the influence of external factors such as contamination or variable feedstock quality that are typical of post-consumer PET. The discussion therefore focuses on correlating changes in molecular structure, rheological behavior, thermal properties, optical appearance, and mechanical performance as a function of cumulative residence time rather than solely the number of processing cycles.

By adopting this framework, the results provide a clear assessment of degradation-driven property evolution and allow the identification of practical limits for the reuse of mechanically recycled PET in bottle-grade as well as lower-demand applications.

### 3.1. Molecular Characteristics

**Figure 2:**
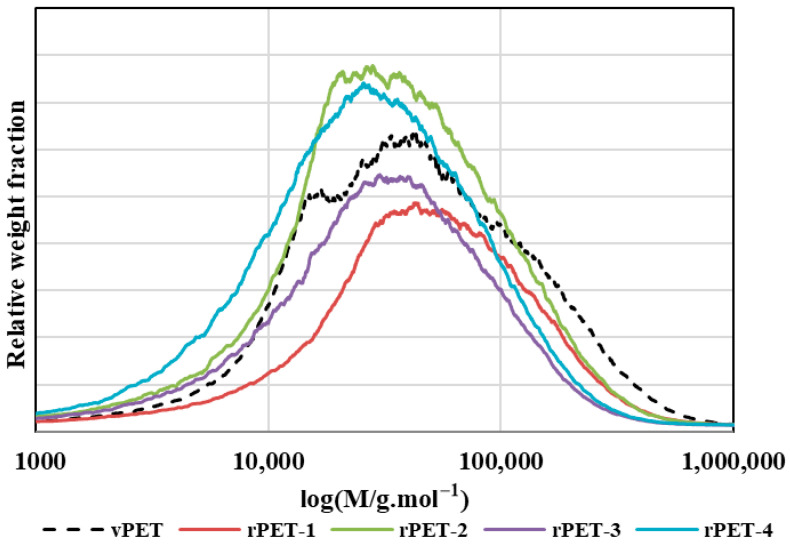
Graphical representation of the molecular weight distribution for PET material subjected to multiple processing cycles using a twin-screw extruder.

Repeated thermo-mechanical processing of PET leads to progressive polymer degradation through chain scission, which is reflected in changes in molecular weight and molecular weight distribution. Gel permeation chromatography (GPC) revealed a systematic shift of the molecular weight distribution toward lower values with increasing extent of processing (Figure 2), indicating gradual shortening of polymer chains during repeated extrusion.

**Figure 3:**
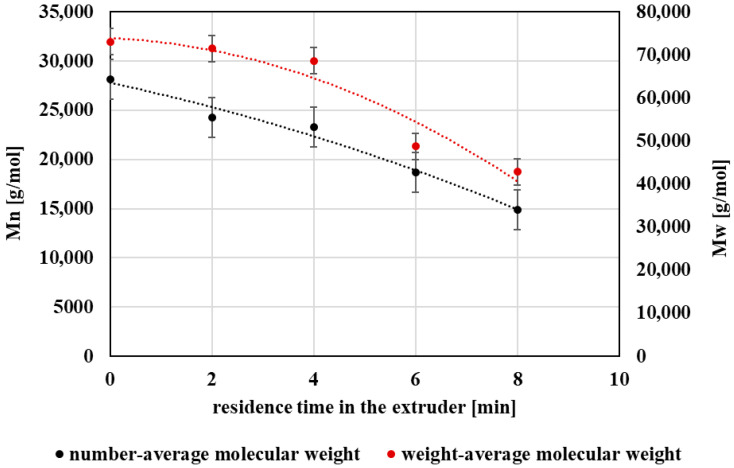
Graphical representation of the number-average molecular weight and the weight-average molecular weight as a function of the residence time of PET material in the processing device.

Both the number-average (Mn) and weight-average (Mw) molecular weights exhibited a pronounced decrease with increasing thermo-mechanical exposure during processing (Figure 3). This trend suggests that the extent of molecular degradation is influenced by a combination of factors, including repeated exposure of the polymer to elevated temperature and shear stresses, while the cumulative residence time in the processing equipment represents one of the relevant parameters contributing to this behavior. The observed reduction in molecular weight is consistent with ester bond cleavage occurring under conditions typical of melt processing.

**Figure 4:**
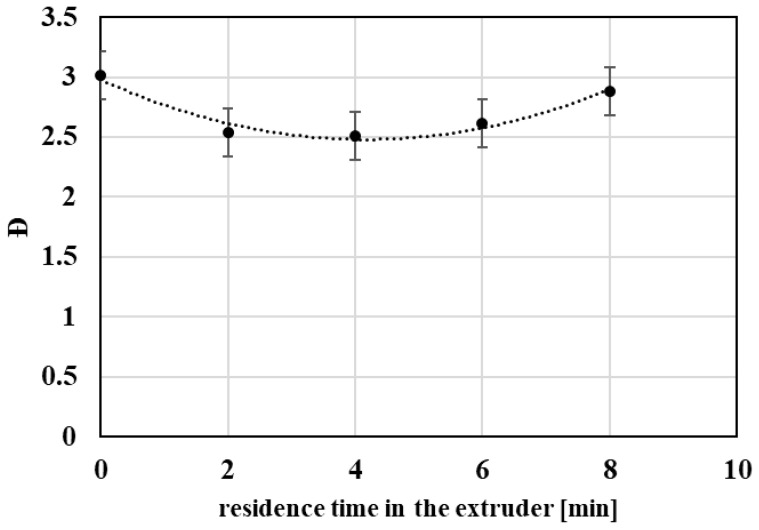
Graphical representation of the polydispersity index as a functions of the residence time of PET material in the processing device.

Changes in molecular weight distribution further support the proposed degradation mechanism. At early stages of repeated processing, a decrease in the polydispersity index (Ð) is observed (Figure 4), indicating preferential scission of the longest polymer chains. At higher degrees of processing, a slight increase in Ð occurs, which may be attributed to more random chain scission affecting a broader range of molecular chain lengths.

The reduction in average molecular weight, together with changes in molecular weight distribution, directly affects the rheological and mechanical behavior of the material, as discussed in subsequent sections. Overall, these results highlight the importance of processing history in assessing the quality and further applicability of mechanically recycled PET.

The observed trends in molecular weight reduction and evolution of molecular weight distribution are in good agreement with previously reported studies on repeated thermo-mechanical processing of PET, where progressive chain scission and preferential degradation of high-molecular-weight fractions were identified as dominant mechanisms [44,47,48]. Similar changes in polydispersity index with increasing processing severity have also been reported in the literature, confirming the general nature of the degradation behavior observed under controlled recycling conditions [51].

### 3.2. Rheological Properties

The rheological behavior of PET is highly sensitive to molecular degradation induced by thermo-mechanical processing, as reflected by changes in intrinsic viscosity (IV) and melt flow rate (MFR). Both parameters provide complementary insight into the evolution of melt behavior during repeated processing and are commonly used as practical indicators of PET processability.

**Figure 5:**
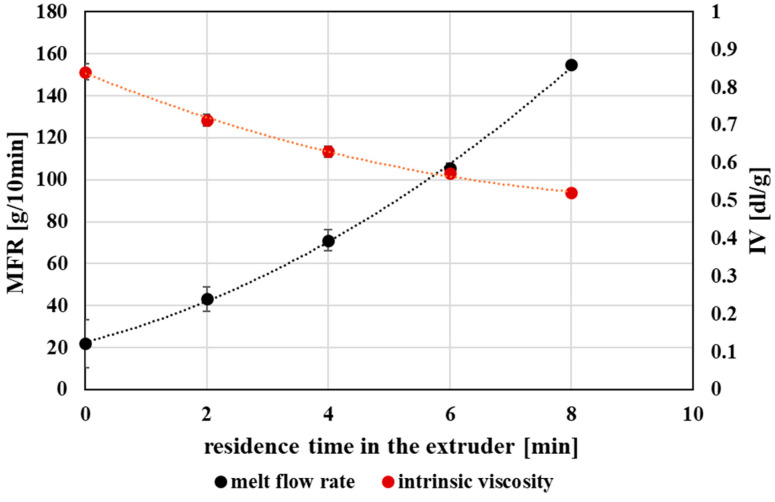
Graphical representation of the intrinsic viscosity and the melt flow index as a function of the residence time of PET material in the processing device.

With increasing extent of processing, a gradual decrease in intrinsic viscosity was observed, accompanied by a pronounced increase in melt flow rate (Figure 5). These trends are consistent with the reduction in molecular weight resulting from chain scission, as shorter polymer chains exhibit lower resistance to flow in the molten state. The inverse relationship between IV and MFR observed in this study is in good agreement with previously reported behavior for mechanically recycled PET.

**Figure 6:**
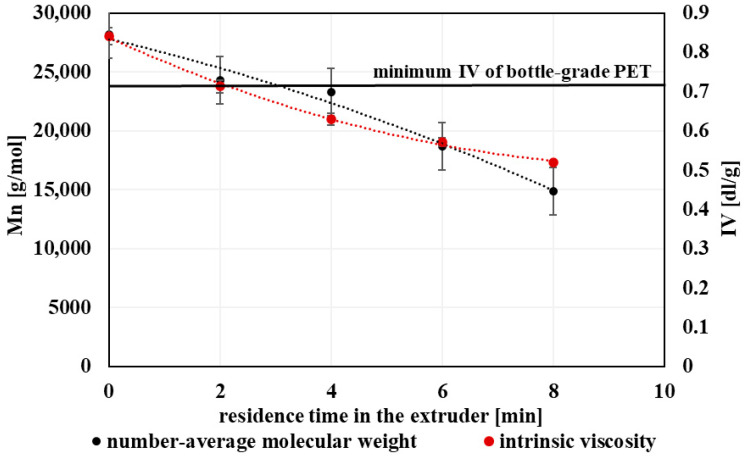
Graphical representation of the number-average molecular weight and the intrinsic viscosity as a function of the residence time of PET material in the processing device.

The observed changes in IV and MFR indicate a progressive loss of melt strength and elasticity with increasing thermo-mechanical exposure. A direct correlation between intrinsic viscosity and molecular weight was confirmed, both decreasing progressively with each recycling cycle (Figure 6). While PET subjected to a limited degree of processing retained rheological characteristics compatible with bottle-grade requirements, further processing led to viscosity levels that may restrict its use in demanding applications requiring high melt strength and dimensional stability.

Nevertheless, the rheological properties of PET processed under higher degrees of thermo-mechanical exposure remain suitable for less demanding applications, such as fibers, films, or non-food packaging, where increased melt flow can be advantageous. Overall, the evolution of IV and MFR highlights the importance of rheological monitoring for assessing the quality and potential reuse pathways of mechanically recycled PET.

The observed increase in melt flow rate and concurrent decrease in intrinsic viscosity with repeated processing are consistent with trends commonly reported for mechanically recycled PET, where chain scission leads to reduced melt viscosity and altered flow behavior [44,47,48].

### 3.3. Thermal Properties

The influence of repeated thermo-mechanical processing on the thermal behavior of PET was evaluated by differential scanning calorimetry (DSC). To minimize the effect of prior thermal history, thermal parameters were determined from the second heating and cooling scans.

**Figure 7:**
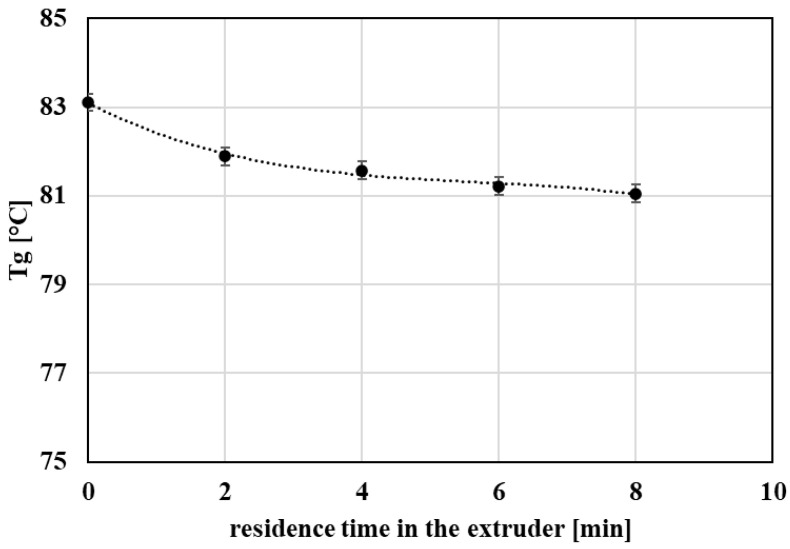
Graphical representation of the glass transition temperature as a function of the residence time of PET material in the processing device.

**Figure 8:**
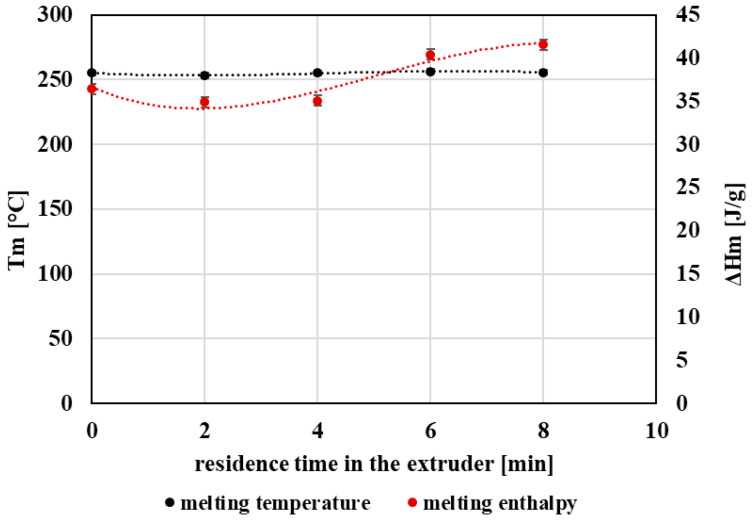
Graphical representation of the melting temperature of crystallites and the melting enthalpy of crystallites as a function of the residence time of PET material in the processing device.

Overall, the glass transition temperature (Tg) exhibited only minor changes with increasing extent of processing (Figure 7), indicating that the amorphous-phase segmental mobility is not strongly altered within the investigated processing window. Similarly, the melting temperature (Tm) remained nearly constant, suggesting that the thermal stability of the crystalline phase is not substantially affected by repeated extrusion (Figure 8).

**Figure 9:**
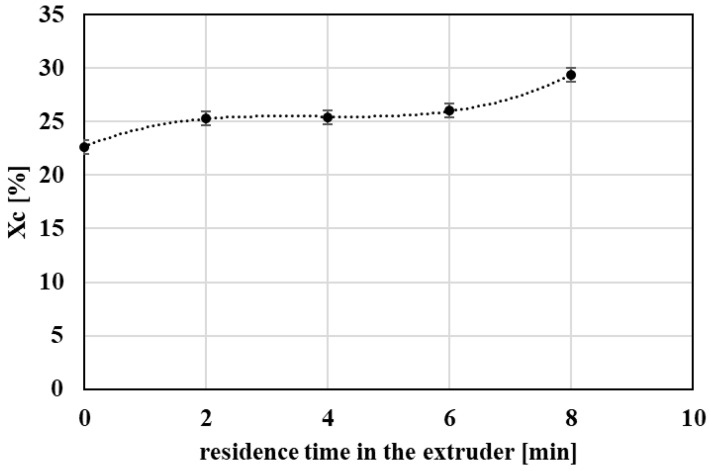
Graphical representation of the degree of crystallinity as a function of the residence time of PET material in the processing device.

**Figure 10:**
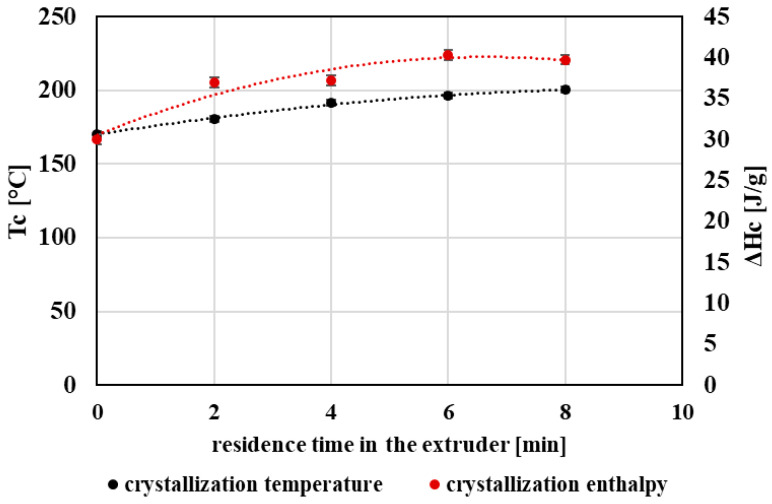
Graphical representation of the crystallization temperature and the crystallization enthalpy during cooling as a function of the residence time of PET material in the processing device.

In contrast, melting enthalpy (ΔHm) (Figure 8) and the degree of crystallinity (Xc) (Figure 9) increased with increasing processing severity. This behavior is consistent with progressive chain scission producing shorter chains with higher mobility, which can reorganize more readily into ordered structures during cooling and heating. Accordingly, the crystallization temperature (Tc) and crystallization enthalpy during cooling also showed an increasing trend (Figure 10), indicating enhanced crystallization tendency after repeated processing.

From a performance perspective, the increase in crystallinity provides a structural explanation for the gradual loss of ductility observed in tensile testing, as a higher crystalline fraction typically reduces the deformability of PET and promotes embrittlement. These results highlight that, while Tg and Tm remain relatively stable, crystallization-related parameters are more sensitive indicators of processing-induced structural changes in mechanically recycled PET.

The increase in crystallinity observed with repeated processing can be attributed to the reduction in molecular weight caused by chain scission, which enhances chain mobility in the molten and supercooled states. Shorter polymer chains are able to reorganize more readily during cooling, leading to more efficient crystal formation without significant changes in the crystalline structure or melting temperature. Similar crystallization behavior of degraded PET has been reported in the literature and is generally associated with enhanced segmental mobility rather than changes in crystal polymorphism [44,48].

### 3.4. Optical Properties—Color Characteristics

Optical properties represent an important indicator of PET quality, particularly in packaging applications where transparency and color stability are critical for material acceptance. Repeated thermo-mechanical processing of PET resulted in measurable changes in color parameters, reflecting ongoing degradation processes within the polymer structure.

**Figure 11:**
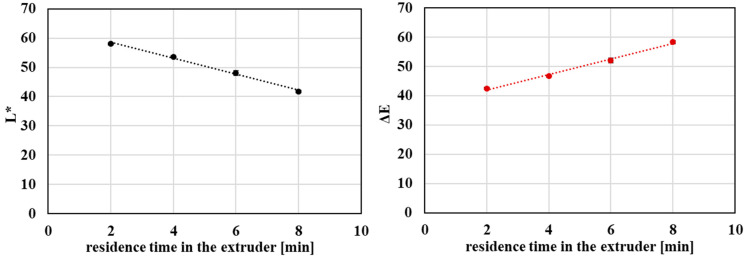
Graphical representation of the lightness and the total color difference as a function of the residence time of PET material in the processing device.

**Figure 12:**
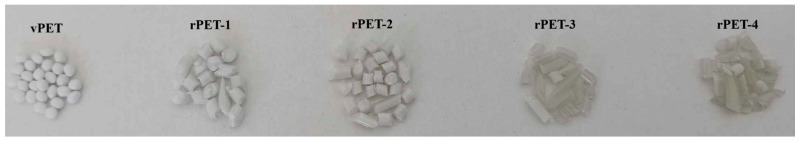
Color change of PET material after each processing cycle.

With increasing extent of processing, a systematic decrease in lightness (L*) accompanied by an increase in total color difference (ΔE) was observed (Figure 11), indicating progressive darkening of the material, which is visible in Figure 12. This behavior is commonly associated with thermo-mechanical degradation of PET, during which the formation of chromophoric structures occurs as a result of ester bond cleavage and subsequent oxidation and condensation reactions.

**Figure 13:**
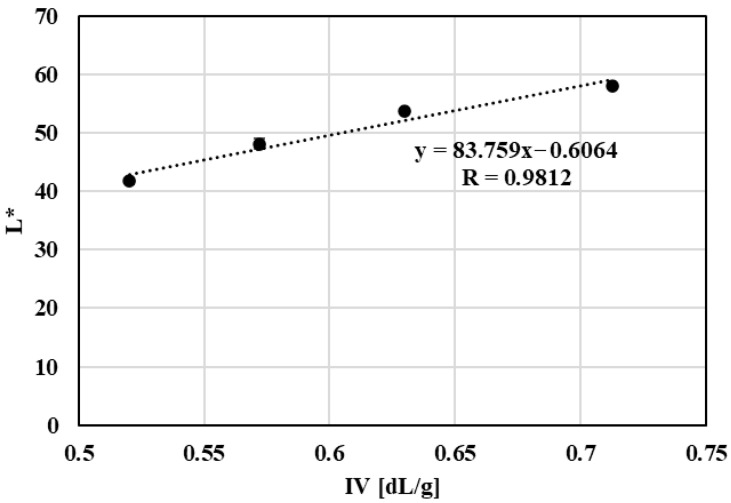
Graphical representation of the lightness as a function of the intrinsic viscosity of PET material.

Analysis of the relationship between optical parameters and intrinsic viscosity revealed a strong linear correlation between lightness (L*) and IV (Figure 13). This correlation was observed under controlled laboratory conditions and should be interpreted as a qualitative indicator rather than a universally predictive relationship. This finding suggests that changes in material lightness are closely linked to progressive molecular degradation of the polymer. Although color changes may be influenced by multiple factors, the observed correlation highlights the potential of optical parameters as a rapid and practical indicator of qualitative changes in PET during mechanical recycling.

From an application perspective, these changes have important implications for the reuse of recycled PET. Increased material darkening limits its suitability for applications requiring high transparency, such as beverage bottles, whereas the material may remain suitable for less demanding applications despite color changes. Overall, the results indicate that optical properties can serve as a sensitive complementary tool for assessing the quality and further applicability of mechanically recycled PET.

### 3.5. Tensile Physical-Mechanical Properties

The influence of repeated thermo-mechanical processing on the mechanical performance of PET was evaluated by tensile testing. Mechanical properties are particularly sensitive to changes in molecular structure and crystalline morphology and therefore provide an important link between molecular degradation and practical material performance.

**Figure 14:**
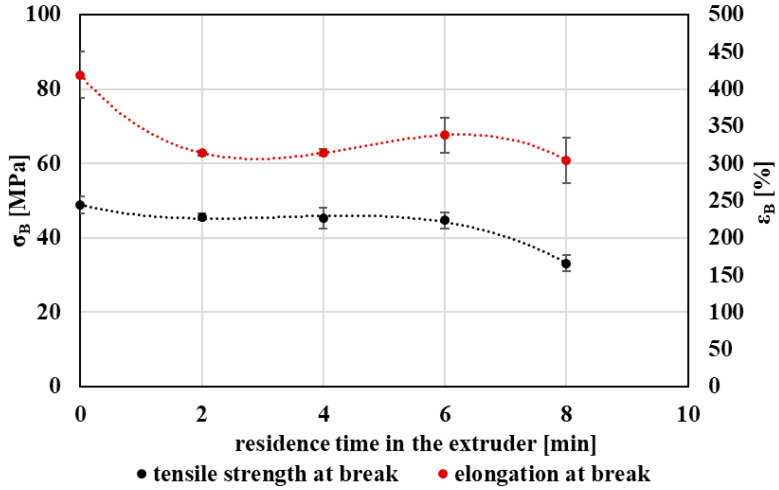
Graphical representation of the tensile strength at break and the elongation at break as a function of the residence time of PET material in the processing device.

With increasing extent of processing, both tensile strength at break and elongation at break exhibited a decreasing trend (Figure 14). This behavior reflects the progressive reduction in molecular weight caused by chain scission, which leads to a loss of chain entanglements and weaker intermolecular interactions, thereby reducing the material’s ability to sustain plastic deformation.

In addition to molecular degradation, the increase in degree of crystallinity observed by DSC contributes to the deterioration of mechanical performance. A higher crystalline fraction generally restricts molecular mobility in amorphous regions, promoting embrittlement and reducing ductility. The combined effects of reduced molecular weight and increased crystallinity therefore provide a consistent explanation for the observed decline in tensile properties with increasing processing severity.

From an application standpoint, PET subjected to a limited degree of thermo-mechanical processing retains mechanical properties compatible with more demanding uses, whereas further processing leads to increased brittleness that may limit its suitability for applications requiring high toughness and elongation. Nevertheless, mechanically recycled PET exhibiting reduced ductility may still be appropriate for less demanding applications, depending on the specific mechanical requirements. Overall, tensile properties confirm the cumulative impact of processing history on the structural integrity and reuse potential of recycled PET. The shape of the stress–strain curves did not indicate pronounced strain hardening prior to fracture, which is consistent with the observed trends in tensile strength and elongation at break.

## 4. Conclusions

This study systematically investigated the effects of repeated thermo-mechanical processing on bottle-grade PET under controlled laboratory conditions, with the aim of isolating intrinsic degradation effects associated with cumulative melt residence time. Mechanical recycling was simulated by successive extrusion passes in a co-rotating twin-screw extruder, enabling a direct correlation between processing history and material property evolution.

Progressive processing led to molecular degradation dominated by chain scission, reflected by a decrease in intrinsic viscosity from approximately 0.80 to 0.65 dL·g^−1^ and a corresponding reduction in molecular weight. While the glass transition temperature and melting temperature remained largely unchanged, crystallization-related parameters were significantly affected. The degree of crystallinity increased from about 23.0% to 29.5%, accompanied by changes in crystallization behavior. These structural changes were associated with a gradual deterioration of mechanical performance, with elongation at break decreasing from approximately 84% to 60%, indicating increased material embrittlement.

The combined reduction in molecular weight and increase in crystallinity provide a consistent physical explanation for the observed loss of ductility and melt strength during repeated processing. In parallel, optical measurements revealed systematic darkening of the material, and a strong correlation between intrinsic viscosity and lightness (L*) was identified, suggesting that optical parameters may serve as a practical complementary indicator of material degradation under controlled conditions.

From an application perspective, the results indicate that bottle-grade PET retains properties compatible with demanding applications only after a limited number of thermo-mechanical processing cycles. Further processing progressively restricts its suitability to less demanding applications, such as fibers, films, and non-food packaging. Environmental factors such as UV exposure and moisture uptake are expected to further accelerate degradation-related changes in properties; however, these effects were beyond the scope of the present study. Overall, this work provides experimentally supported limits for the reuse of mechanically recycled PET and highlights the importance of controlling processing history when defining realistic circular economy pathways.

## Figures and Tables

**Table 1:** Designation of the tested samples for the multiple recycling process.

Sample Designation	Processing	Processing Time (min.)
vPET	virgin PET	0
rPET-1	1st processing cycle	2
rPET-2	2nd processing cycle	4
rPET-3	3rd processing cycle	6
rPET-4	4th processing cycle	8

**Table 2:** Conditions for measuring thermal properties.

	Ramp	Set Temperature (°C)	Time (min)
Conditioning	isothermal	20	1
1st Heating	10 °C/min.	300	28
Conditioning	isothermal	300	1
Cooling	10 °C/min.	0	28
Conditioning	isothermal	0	1
2nd Heating	10 °C/min.	300	28

## Data Availability

The original contributions presented in this study are included in the article. Further inquiries can be directed to the corresponding author.
